# A genetic algorithm for the arrival probability in the stochastic networks

**DOI:** 10.1186/s40064-016-2265-7

**Published:** 2016-05-20

**Authors:** Gholam H. Shirdel, Mohsen Abdolhosseinzadeh

**Affiliations:** Department of Mathematics, Faculty of Basic Science, University of Qom, Qom, Iran

**Keywords:** Stochastic network, Genetic algorithm, Discrete time Markov chain, Arrival probability, Primary 90C59, 90C35; Secondary 90C40

## Abstract

A genetic algorithm is presented to find the arrival probability in a directed acyclic network with stochastic parameters, that gives more reliability of transmission flow in delay sensitive networks. Some sub-networks are extracted from the original network, and a connection is established between the original source node and the original destination node by randomly selecting some local source and the local destination nodes. The connections are sorted according to their arrival probabilities and the best established connection is determined with the maximum arrival probability. There is an established discrete time Markov chain in the network. The arrival probability to a given destination node from a given source node in the network is defined as the multi-step transition probability of the absorbtion in the final state of the established Markov chain. The proposed method is applicable on large stochastic networks, where the previous methods were not. The effectiveness of the proposed method is illustrated by some numerical results with perfect fitness values of the proposed genetic algorithm.

## Background

Established connections in networks should be reliable to transmit flow from a source node to a destination node especially in delay sensitive networks. Determination of the best connection is one of the most important problems to avoid traffic congestion in networks. So, the arrival probability is used to evaluate the reliability of an established connection and it has been considered as an optimality index of the stochastic shortest path length (Bertsekas and Tsitsiklis 1991; Fan et al. 2005; Kulkarni 1986). The stochastic shortest path problem (SSP) is defined in a network with stochastic parameters. Liu (2010) produced the SSP where arc lengths were assumed to be uncertain variables. Pattanamekar et al. (2003) considered the individual travel time variance and the mean travel time forecasting error. Also, Hutson and Shier (2009) and Rasteiro and Anjo (2004) supposed two criteria, mean and variance of path length. Fan et al. (2005) assumed that each link to be congested or uncongested, with known conditional probabilities for link travel times. Wu et al. (2004) modeled a stochastic and time-dependent network with discrete probability distributed arc weights. The considered model in this paper is a directed acyclic stochastic network with known discrete distribution probabilities of leaving or waiting in nodes.

Our criterion to evaluation of the connections from the source node toward the destination node in the network is presented as the arrival probability, which is obtained by the established discrete time Markov chain (DTMC) in the network. Liu (2010) applied models according to the decision criteria and converted them into deterministic programming problems. Hutson and Shier (2009) and Rasteiro and Anjo (2004) determined the path with maximum expected value of a utility function. Fan et al. (2005) proposed a procedure for dynamic routing policies. Nie and Fan (2006) formulated the stochastic on-time arrival problem with dynamic programming, and Fan et al. (2005) minimized the expected travel time. In this paper, the maximum arrival probability from a given source node to a given destination node is computed according to known discrete distribution probabilities of leaving or waiting in nodes, and a DTMC stochastic process is used to model the problem rather than dynamic programming or stochastic programming.

Kulkarni (1986) developed a method based on a continuous time Markov chain (CTMC) to compute the distribution function of the shortest path length. Azaron and Modarres (2005) applied Kulkarni’s method to queuing networks. Thomas and White (2007) modeled the problem of constructing a minimum expected total cost route as a Markov decision process. They wanted to respond to dissipated congestion over time according to some known probability distribution. From the static viewpoint, our studied model is related to the dynamic time-evolving networks recently studied by Shang (2015a, b), however problems addressed are from a different field.

The stochastic topology of networks motivated us to consider the arrival probability from the source node toward the destination node. So, the arcs of the network could be congested probably, that it is commonly happen in communication and transportation networks where the connecting arcs of some nodes are unable to transmit flow. The stochastic topology of the network causes several unstable connections between nodes; however, the physical topology of the network determines possible and impossible connections between pairs of nodes. The leaving distribution probability from one node toward another node is known as the probability that their connected arc to be uncongested. A DTMC with an absorbing state is established and the transition matrix is obtained. Two conditions at any state of the established DTMC are assumed: departing from the current state to a new state when a larger labeled node is visited in the original network, or waiting in the current state with expecting better conditions. Then, the probability of arrival the destination node from the source node is computed as the multi-step transition probability from the initial state to the absorbing state in DTMC.

The proposed method applies DTMC and a genetic algorithm is produced to obtain an acceptable solution, that it applies small locally created state spaces instead of the original large state space. The computed arrival probability describes the overall situation of the network to transmit flow from the source toward the destination; while, the previous works focused on a specific path Liu (2010), Hutson and Shier (2009), Rasteiro and Anjo (2004).

The remain of the paper is organized as follows. Section “The stochastic topology of the network” consists of some preliminary definitions and assumptions for the considered model of the stochastic network. The established DTMC and computational method for the arrival probability is presented in “The established discrete time Markov chain” section. In “A genetic algorithm to find the arrival probability” section, a genetic algorithm is presented to approximate the arrival probability. In section “Numerical results”, some implementations of the proposed method on the networks with large size of nodes and arcs are provided.

## The stochastic topology of the network

Directed acyclic networks are considered for various applications, some of typical examples are related in the following: citation networks in information sciences, phylogenetic networks in biology, data structures in computer science and engineering, acyclic graphs in pure mathematics, random graphs and Bayesian networks in statistics, and etc. (for more details see Ahuja et al. 1993; Karrer and Newman 2009). Recently, Shang (2014) considered group consensus problems in generic linear multi-agent systems with directed information flow under directed fixed interaction topology and randomly switching, where the underlying networks are governed by a continuous-time Markov process.

Let network $G=(N,A)$, with node set *N* and arc set *A*, be a directed acyclic network. Then, we can label nodes in a topological order such that for any $(i,j)\in A$, $i<j$ (Ahuja et al. 1993). The physical topology for any $(i,j)\in A$ shows the connection of nodes $i,j\in N$. Actually, the physical topology shows the possibility of communication between nodes in the network. To model the stochastic topology of a network think about the transportation networks, where there are some physical connections between nodes but we cannot traverse anymore toward the destination node because of probable congestion. Network *G* has a stochastic topology if there are some facilities in the network but it is not possible to use them continuously. So, the existence of any arc $(i,j)\in A$ does not mean there is a stable communication between nodes $i,j\in N$ all the time (it could be probably congested). For any node *i*, it is supposed that the uniform distribution probabilities of leaving arcs (*i*, *j*) to be uncongested are known.

Now, consider the situation that flow arrives in a node but cannot leave because of the stochastic topology (some arcs are congested), then waits for better conditions. There are two options for wait situations: first, waiting in a particular node with expecting some facilities to release from the current situation, which is called option 1, second, traversing some arcs those do not lead to visit a new node, which is called option 2. The stochastic variable of arc (*i*, *j*) according to a stochastic topology is shown by $x_{ij}$. If $x_{ij}=1$, it is possible to traverse arc (*i*, *j*) and otherwise $x_{ij}=0$. The probability that arc (*i*, *j*) to be uncongested is $q_{ij}=Pr[x_{ij}=1]$. Then, the wait probability in node *i*, is $q_{ii}=1-\sum _{\{j:(i,j)\in A\}}q_{ij}$.

**Fig. 1:**
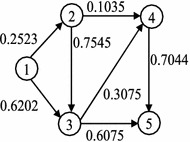
The example network with 5 nodes and 7 arcs

Figure 1 shows the example network with its topological ordered nodes and it is the initial physical topology of the network. The numbers on arcs show the leaving probabilities $q_{ij}$. Node 1 is the source node and node 5 is the destination node. It is not possible to traverse arc (1, 4) because it does not exist in the physical topology of the example network. However, the arcs in the physical topology could be congested according to the known distribution probabilities.

## The established discrete time Markov chain

Kulkarni (1986), Azaron and Modarres (2005) considered an acyclic directed network. They produced a CTMC and in each transition from one state to another possibly more than one node can be added. Then, they computed the length of the shortest path with exponentially distributed arc length. We propose a DTMC with discrete distributed probabilities, such that in each transition only one node can be added, and the wait states are more extended. The discrete time stochastic process $\{X_r,r=1,2,3,...\}$ is called Markov chain, if it satisfies the following Markov property (see Ross 2006 and Thomas and White 2007)

$$\begin{aligned} Pr[X_{r+1}=S_l|X_r=S_k,X_{r-1}=S_m,...,X_1=S_n]=Pr[X_{r+1}=S_l|X_r=S_k]=p_{kl}. \end{aligned}$$

Any state $S_l$ of the established DTMC determines the traversed nodes of the original network. For the example network (Fig. 1) the created states $S_i$, are shown in Table 1. The conditional probability of next state depends on the current state and independent of the previous states. Let $S=\{S_i,i=1,2,3,...\}$, the initial state $S_1=\{1\}$ of DTMC contains the single source node and the absorbing state $S_{|S|}=\{1,2,...,|N|\}$ contains all nodes of the network and it is not possible to depart; so, *S* is a finite state space (it is not possible departing from $S_{|S|}$).

**Table 1:** The state space of the example network

State space	Current nodes
$S_1$	{1}
$S_2$	{1, 2}
$S_3$	{1, 3}
$S_4$	{1, 2, 3}
$S_5$	{1, 2, 4}
$S_6$	{1, 3, 4}
$S_7$	{1, 2, 3, 4}
$S_8$	{1, 2, 3, 4, 5}

**Fig. 2:**
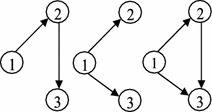
Constructed connected components of state $S_{4}$

For the example network, the absorbing state $S_8=\{1,2,3,4,5\}$ contains all nodes of the network; and the instance state $S_4$ of the state space *S* (Table 1) contains nodes $\{ 1,2,3\}$ and all connected components of the example network, those are constructed by nodes 1, 2 and 3, see Fig. 2.

The final state contains the destination node of the network, where DTMC does not progress anymore (Assumption 1). The states of the established DTMC contain the traversed nodes of the network, those are reached from some nodes in a previous state (Assumption 2). It is not allowed to return from the last traversed node, however it is possible to wait in the current state. Clearly, a new state is revealed if a leaving arc $(i,j)\in A$ is traversed such that the current node *i* is contained in the current state and the new node *j* is contained in the new state (Assumption 3). As previously said, the wait states are one of option 1 or option 2. So, following assumptions describe construction of the state space of the established DTMC:

1.: By arriving the destination node, the process can traverse neither any node nor any arc (i.e. the absorbing state)
2.: The new state is created, if a new node is added to the current state nodes
3.: According to the current state, it is allowed to reach only one node during transition to a new state.

**Fig. 3:**
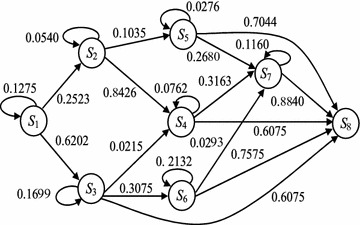
The state space diagram of the established DTMC

Finally, by Assumptions 1, 2 and 3 all possibilities of transmission flow from the source node toward the destination node in the network are examined dynamically. The state space diagram of the established DTMC for the example network is constructed as Fig. 3; the values on arcs show the wait and the transition probabilities.

### The transition and the wait probabilities

The transition probabilities $p_{kl}$ satisfy the following conditions

- $$0\le p_{kl}\le 1\,\,$$ for $$k=1,2,...,|S|$$ and $$l=1,2,...,|S|$$
- $$\sum _l{p_{kl}}=1\,\,$$, for $$k=1,2,...,|S|$$.

The transition probabilities are elements of matrix $P_{|S|\times |S|}$, where $p_{kl}$ is *k*th row and *l*th column of matrix *P*, and it is called the transition matrix or Markov matrix (Ibe 2009). The transition matrix of the established DTMC in the network is obtained by following theorems. The transition probabilities (except to the absorbing state) are obtained by Theorem 1.

#### **Theorem 1**

*If*$p_{kl}$*isklelement of matrix P, that*$k \ne l$, $l < |S|$*and*$S_{k}=\{v_{0}=1,...,v_{m}\}$*is thecurrent state, then the transition probability from state*$S_{k}$*to state*$S_{l}$*is computed as follow*

*If*$l < k$*then*$p_{kl} = 0$, *otherwise if*$l > k$*then*

$$\begin{aligned} p_{kl}=Pr\left[\bigcup _{\left( v,w\right) \in \Psi }{E_{vw}} \right]\times \left(\prod _{\left( v,w\right) \in \Psi } \left(1-\sum _{\begin{array}{c} \left( v,u\right) \in A \\ u\ne w,u\notin S_k \end{array}} q_{vu} \right)\right)\times q_{v_mv_m}+q_{v_mw}. \end{aligned}$$

$E_{vw}$*denotes the event which arc* (*v*, *w*) *is traversed during transition from*$S_k$*to*$S_l$*and*$\Psi =\{(v,w)\in A:v\in S_k\backslash \{v_m\},w\in S_{l}\backslash S_k,|S_l\backslash S_k|=1\}$.

#### *Proof*

Since, it is not allowed to traverse from one state to the previous states (Assumption 2), then necessarily $p_{kl}=0$, for $l\ <\ k$. Otherwise, suppose $l\ >\ k$, during transition from the current state $S_k$ to the new state $S_l$, it should be reached just one node other than the nodes of the current state, so $|S_l\backslash S_k|=1$, $v\in S_k$, and $w\in S_l\backslash S_k$ are held by Assumptions 2 and 3. Two components of $p_{kl}$ formula should be computed.

In the last node $v_m$ of the current state $S_k$, it is possible to wait in $v_m$ with probability $q_{v_mv_m}$. Notice, it is not possible to wait in the other nodes $v\in S_k\backslash \{v_m\}$ because it should be leaved to construct the current state, however it is not necessary for node $v_m$ with the largest label (leaving $v_m$ leads to a new node, and therefore results in a new state). If $w\in S_l\backslash S_k$, then one or all of events $E_{vw}$ (i.e. traversing a connecting arc between a node of the current state and another node of the new state) can happen for $\left( v,w\right) \in \Psi$, and the arrival probability of node $w \in S_l$ from the current state $S_k$ is equal to $Pr[\bigcup _{\left( v,w\right) \in \Psi }{E_{vw}}]$. The collection probability should be computed because of deferent representations of the new state (for example see Fig. 2). Then, the nodes of the current state $v\in S_k\backslash \{v_m\}$ (while waiting in $v_m$) should be prevented from reaching other nodes $u\notin S_k$ and $u\ne w$ (Assumption 3), so arcs $\left( v,u\right)$ are not allowed to traverse and they are excluded simultaneously, thus it is equal to $$\prod _{\left( v,w\right) \in \Psi }{(1-\sum _{\begin{array}{c} \left( v,u\right) \in A \\ u\ne w,u\notin S_k \end{array}}{q_{vu}))}}$$. The other possibility in node $v_m$ that is leaving it toward the new node $w\in S_l\backslash S_k$ with probability $q_{v_mw}$. $\square$

**Fig. 4:**
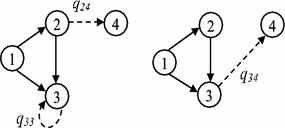
The constructed states during transition from $S_4$ to $S_7$

For example, in the established DTMC of the example network, the transition probability $p_{47}$ is computed by the constructed components as shown in Fig. 4; and it is $Pr(E_{14}\cup E_{24})\times (1-q_{15})(1-q_{25})\times q_{33}+q_{34}$, where $Pr(E_{14}\cup E_{24})= q_{14}+q_{24}-q_{14}q_{24}$, however $q_{14}=q_{15}=q_{25}=0$ as shown in Fig. 1, then $p_{47}=q_{33}\times q_{24}+q_{34}$. It is possible to wait in node 3 but not other nodes of the current state $S_4=\{1,2,3\}$; where, by traversing arc (2, 4) or (3, 4) the new state $S_7=\{1,2,3,4\}$ is revealed.

Theorem 2 describes the transition probabilities to the absorbing state $S_{|S|}$, and they are the last column of the transition matrix *P*.

#### **Theorem 2**

*To compute the transition probability from state*$S_k=\{v_0=1,...,v_m\}$* to the absorbing state*$S_{|S|}$*for*$k=1,2,...,|S|-1$*, which is k*|*S*| *th element of matrix P*, *suppose*$v_n\in S_{|S|}$*is the given destination node of the network then*

$$\begin{aligned} p_{k|S|}=Pr\left[\bigcup _{v\in S_k,(v,v_n)\in A}E_{vv_n}\right]. \end{aligned}$$

$E_{vv_n}$*denotes the event that arc*$(v,v_n)\in A$*of the network is traversed during the transition from*$S_k$*to*$S_{|S|}.$

#### *Proof*

To compute the transition probabilities $p_{k|S|}$, for $k=1,2,...,|S|-1$ it should be noticed the final state is the absorbing state $S_{|S|}=\{1,2,3,...,|N|\}$ containing all nodes of the network, and the stochastic process does not progress any more (Assumption 1). So, it is sufficient to consider leaving arcs $(v,v_n)$ from $v\in S_k$, the nodes of the current state, toward the destination node $v_n\in S_{|S|}$. Then, one or all of events $E_{vv_n}$ (i.e. traversing a connecting arc between a node of the current state and the destination node of the absorbing state) can happen and the transition probability from the current state $S_k$ to the absorbing state $S_{|S|}$ is totally equal to $Pr[\bigcup _{v\in S_k,(v,v_n)\in A}E_{vv_n}]$. The collection probability should be computed because of deferent representations of the states (for example see Fig. 2). $\square$

For state $S_4$, transition probability $p_{48}$ is obtained by $Pr(E_{15}\cup E_{25}\cup E_{35})$, however $q_{15}=q_{25}=0$, then $p_{48}=q_{35}$. The wait probabilities, those are the diagonal elements of the transition matrix *P*, are obtained by Theorem 3.

#### **Theorem 3**

*Suppose*$S_k=\{v_0=1,...,v_m\}$*is the current state, then the wait probability*$p_{kk}$*is kth element of matrix P and it is*

$$\begin{aligned} p_{kk}= {\left\{ \begin{array}{ll} 1-\sum ^{|S|}_{j=k+1}p_{kj} & \quad {\text {if}}\,\, k<|S| \\ 1 &\quad {\text {if}}\,\, k=|S|. \end{array}\right. } \end{aligned}$$

#### *Proof*

The wait probabilities $p_{kk}$ are the complement probabilities of the transition probabilities from the current state $S_k$, for $k=1,2,...,|S|-1$, toward the all departure states $S_j$, for $j=k+1,k+2,...,|S|$. Then, we have $p_{kk}=1-\sum ^{|S|}_{j=k+1}p_{kj}$, for $k=1,2,...,|S|-1$, in other word, they are the diagonal elements of matrix *P*, those are computed for any row $k=1,2,...,|S|-1$ of the transition matrix (see Ibe 2009). The absorbing state $S_{|S|}$ does not have any departure state, so $p_{|S||S|}=1$ as the transition matrix *P*.□

### The arrival probability

The arrival probability determines the reliability of connections in the network, and it shows the probability that they are not congested during transmission flow from the source node to the destination node in the network. The arrival probability is defined as multi-step transition probability from the initial state $S_1$ to the absorbing state $S_{|S|}$ in the established DTMC. According to Assumptions 1, 2 and 3, the state space of DTMC is directed and acyclic (otherwise return to the previous states is allowed). Out-degree of any state is at least one (without loop wait transition arcs consideration), except the absorbing state $S_{|S|}$, then for any state $S_k$, there is one$\setminus$multi-step transition from the initial state to the absorbing state that traverses state $S_k$. Consequently, the absorbing state is accessible from the initial state after some finite transitions. Let $p_{kl}(r)=Pr[X_{m+r}=S_l|X_m=S_k]$ denote the conditional probability that the process will be in state $S_l$ after exactly *r* transitions, given that it is presently in state $S_k$. So, if matrix *P*(*r*) is the transition matrix after exactly *r* transitions, it can be shown that $P(r)=P^r$, and let $p_{kl}(r)$ be *kl*th element in matrix $P^r$ (see Ibe 2009). Thus, the arrival probability after exactly *r* transitions is $p_{1|S|}(r)=Pr[X_r=S_{|S|}|X_0=S_1]$ and it is the 1|*S*|th element in the matrix $P^r$.

**Fig. 5:**
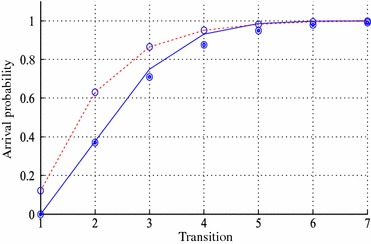
The arrival probabilities

For the example network, we want to obtain the probability of the arrival node 5 from node 1. The arrival probability $p_{18}(r)$ is obtained as shown by solid line in Fig. 5 after seven transitions. For *r* sufficiently large, the probabilistic behavior of DTMC becomes independent of the starting state i.e. $Pr[X_r=S_{|S|}|X_0=S_1]=Pr[X_r=S_{|S|}]$, that is the multi-step transition probability (Ibe 2009). For the example network, it is 0.9994.

## A genetic algorithm to find the arrival probability

Although it has been shown Markov decision problems could be solved in polynomial time (Papadimitriouc and Tsitsiklis 1987), computations grow exponentially in practice (Littman et al. 1995). The proposed DTMC is very sensitive to the size of the networks (specially the size of the node set), and its constructed state space is grown exponentially (see last column in Table 2). Then, a genetic algorithm is produced to obtain a near optimal solution. So, it is a polynomial time algorithm since it is done over the established DTMC, which has polynomial computation complexity. However, the proposed genetic algorithm applies small locally created state spaces instead of the gigantic large state space.

**Table 2:** The implementation of the proposed genetic algorithm

Node number	Arc number	Transition component size	Almost value	Fitness probability	Arrival space size	State
	20	5		0.9981	0.9771	127
10	26	5	9	1.0000	0.9977	130
	29	5		0.9902	0.9249	253
	82	15		0.9973	0.9973	126,593
20	98	15	13	0.9998	0.9998	200,593
	105	15		0.9999	0.9947	122,385
	208	22		0.9999	0.8492	
30	216	22	13	1.0000	0.9623	
	228	22		1.0000	0.9488	
	363	40		1.0000	0.8927	
40	372	60	11	1.0000	0.9754	
	386	60		1.0000	0.7571	
	609	87		1.0000	0.9719	
50	614	87	11	1.0000	0.8559	
	630	75		1.0000	0.6983	

The proposed genetic algorithm needs to obtain some initial feasible solutions, so the first step is to extract some sub-networks from the original network with local source nodes and destination nodes. Extracting the sub-networks is applied by all operators of the proposed genetic algorithm and it is presented as a basic operator. We need to define three types of operators as described by Sivanandam and Deepa (2008) and Dréo et al. (2006). The initial population operator extracts some sub-networks and their required complement components to establish connections between the original source and destination nodes. Then, the crossover operator tries to obtain new populations from the current initial populations and to replace the worst ones with the best ones. To avoid probable local optimality, the mutation operator extends the search area of selecting local source and destination nodes. Therefore, the sub-networks inherit all characteristics of the original network; they are directed, acyclic and all nodes are reachable from the local source node and the local destination node is reachable form all nodes. A connection is a union of some sub-networks, those the local source node of one is the local destination node of another one and it can transmit flow from the original source node toward the original destination node.

### Extracting a sub-network from the original network

Any sub-network from the original network needs a source node that sends flow faster than those nodes their labels are smaller and it should be the smallest index that could be departed from. Also, it needs a destination node which receives flow faster than those nodes their labels are grater and it should be the greatest index that could be arrived. So, consider the following situations:

- If node *i* is a local source node for a sub-network, then the leaving probabilities are changed as below
  1 $$\begin{aligned} \forall v<i,\quad \forall w\in N: {\left\{ \begin{array}{ll} q_{vv}:=q_{vv}+q_{vw},&\quad q_{vw}:=0\\ q_{ww}:=q_{ww}+q_{wv},&\quad q_{wv}:=0. \end{array}\right. } \end{aligned}$$
- If node *j* is a local destination node for a sub-network, then the weights of arcs are changed as below
  2 $$\begin{aligned} \forall v>j, \quad \forall w\in N: {\left\{ \begin{array}{ll} q_{vv}:=q_{vv}+q_{vw},&\quad q_{vw}:=0\\ q_{ww}:=q_{ww}+q_{wv},&\quad q_{wv}:=0. \end{array}\right. } \end{aligned}$$

By (1) and (2) we will be able to separate a component of the network; however, there maybe exist some nodes except the local source node with in-degree zero, or some nodes except the local destination node with out-degree zero, they could be determined from the changed arc weight matrix. The node set and the new arc weight matrix for a sub-network are generated as below:

#### Extracting sub-network operator

1. For any node *v* satisfying (1) and (2) omit node *v* from the node set and row *v* and column *v* form the arc weight matrix.
2. While there exists any zero row *k* (column *k*) except the row and the column of the local destination node, omit node *k* from the current node set and row *k* and column *k* from the current arc weight matrix.

Therefore, the operator I guaranties existence of a local source node and a local destination node, and the operator II guaranties connectivity. So, if the local destination node is unreachable from the local source node, then the final arc weight matrix will be an identity matrix of size $(|N|-1)\times (|N|-1)$, and the current local source and destination nodes should be changed and the extracting operator is repeated. At the end of the extracting operator, for any node *i* there exists a path from the local source node to any node *i* of the extracted sub-network, as well as a path from any node *i* of the extracted network to the local destination node; otherwise, there is a contradiction with the original network characteristics. Except the first column and the last row, there are no zero columns or rows in the weight matrix of the sub-network (the wait probabilities are replaced with the zero diagonal elements of the matrix). For the example network, we suppose the constructed components of the sub-networks have at most three nodes.

### The initial population operator

To accomplish a connection between the original source and destination nodes, it is required to produce some complement components. Obviously, there should exist such components those could connect together, otherwise there is not feasible solution for the problem. The proposed genetic algorithm starts with some randomly created initial components; then, mid-components are obtained such that a connection between the original source node and the original destination node is established. For producing complement components, the local destination node of a sub-network and the local source node of another one are selected according to their labels, then a mid-component is created with these successive local source and destination nodes; especially, the original source node and the original destination node are considered as components themselves.

#### The initial population operator

1. Choose a local source node and a local destination node randomly.
2. Extract the sub-network with local source and destination nodes (initial component).
3. Put the initial local source node as $s_0$, and the initial local destination node as $d_0$.
4. Create a mid-component between the original source node and $s_0$: put $s_0$ as the local destination node, then select randomly the local source node *i* for $i<\ s_0$; if $i\ne s$, then put $s_0=i$ and repeat.
5. Create a mid-component between $d_0$ and the original destination node: put $d_0$ as the local source node, then select randomly the local destination node *i* for $i>\ d_0$; if $i\ne d$, then put $d_0=i$ and repeat.

**Fig. 6:**
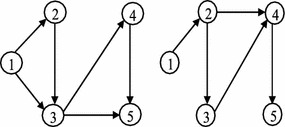
The created connections of the extracted sub-networks

The connections are sorted according to their fitness values from the worst one to the best one. The fitness value shows the arrival probability from the original source node to the original destination node (see “The arrival probability” section). The initial population operator is ended when the number of the initial populations is satisfied. The implementation of the initial population operator on the extracted sub-networks of the example network creates connections as shown in Fig. 6.

### The crossover operator

To produce new populations of the current populations and to improve the current optimality, we define the crossover operator similar to the initial population operator, except that the local source and the destination are selected form the nodes belonging to sub-networks. To produce distinct populations, the algorithm chooses one node from the set $\pi _1=\{\text {the nodes belonging to the initial population}\ i\}-\{\text {the nodes belonging to the initial population}\ j\}$ and another node from the set $\pi _2=\{\hbox {the nodes belonging to the initial population } j\}-\{\hbox {the nodes belonging to the initial population }i\}, i\ne j$, if none of them is empty; otherwise, one of the obtained initial populations is the sub-set of the other one and the algorithm extends the search area by the mutation operator.

#### The crossover operator

1. If none of sets $\pi _1$ and $\pi _2$ is empty then randomly choose one node belonging to $\pi _1$ and one node belonging to $\pi _2$, as the local source node and the local destination node, according to their labels.
2. Extract a sub-network and apply the initial population operator on the new extracted sub-networks.

### The mutation operator

To avoid local optimality the mutation operator extends the search area to other parts of the network. So, if the current populations do not change during the crossover operator (one of sets $\pi _1$ and $\pi _2$ is empty), the mutation operator extends the search area of the local source and destination nodes detection, and it tries to change the current populations and improves the arrival probability.

#### The mutation operator

1. Select two nodes randomly belonging to $N-(\{\hbox {initial population }i\}\bigcup \{\hbox {initial population}\, j\})$ as the local source node and the local destination node.
2. Extract a sub-network and apply the initial population operator on the new extracted sub-networks.

### Fitness function

The selection operator through the algorithm is a ranked selection operator. Connections are sorted from the worst one to the best one, then the proposed genetic algorithm replaces the worst one with the new connections of the improved fitness values. After the multi-step transition probabilities of the all components contained in any population were computed, a path is constructed between the original source node and the original destination node through the local destination nodes of the initial population. Then, by the similar process to the original network, the arrival probability is computed for the path and it is recorded as the fitness value of the population. In any iteration, the above process is repeated for the optimal populations with the maximum arrival probabilities. Out-put of the proposed genetic algorithm for the example network is shown by dash line in Fig. 5. The obtained arrival probability is 0.9975 and it is given after 7 transitions, with 5 initial populations and its fitness value is 1.0000.

## Numerical results

Some implementations of the proposed method on large networks are presented in this section (see Table 2). The networks are acyclic directed networks and there is a path from each node to the destination node. The leaving and waiting probabilities of nodes are random numbers produced by the uniform distribution probability. Then, the arrival probability is computed for the established DTMC. All of the experiments are coded in MATLAB R2008a and they are performed on Dell Latitude E5500 (Intel(R) Core(TM) 2 Duo CPU 2.53 GHz, 1 GB memory).

Any increment in the number of nodes and arcs of the network increases the state space size, consequently. So, the transition matrix and its related computations need gigantic amount of memory (see last column of Table 2) such that it is not easy to find the exact solution. For networks with 10 nodes, the size of the state space is about hundreds, for networks with 20 nodes it is about thousands, and for networks with 30 nodes it is about millions, and so on. Third column of Table 2 shows required nodes to create the sub-networks and the arrival probabilities are obtained with transition numbers.

**Fig. 7:**
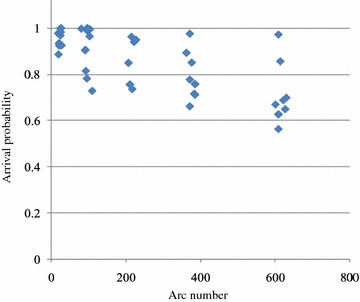
The out-put of the genetic algorithm according to arc number

The implementations of the proposed genetic algorithm according to the arc numbers are shown in Fig. 7. Results show the numbers of the components containing in the constructed sub-networks and the numbers of transitions, both are two most important parameters to obtain the arrival probability by the proposed genetic algorithm.

## Conclusion

We considered an established discrete time Markov chain stochastic process over directed acyclic networks. The arrival probability from a given source node to a given destination node was computed according to the probability of transition from the initial state to the absorbing state by multi-step transition probability in DTMC. A genetic algorithm was proposed for large networks, where the state space of the established DTMC grew as rapidly as exponentially. However, the proposed genetic algorithm applied small locally created state spaces instead of the gigantic large state space. Numerical results showed efficiency of the proposed method to obtain the multi-step transition probability that the destination node is accessible for the first time. Extension of described model on the continuous time varying networks, and using the discrete nature of the proposed model to apply meta-heuristic methods and reducing the computations can be considered as future works guidelines. Also, the shortest path problem with recourse where some local decisions are made during routing process and they could be evaluated by the proposed method.
